# VDAC1 Conversely Correlates with Cytc Expression and Predicts Poor Prognosis in Human Breast Cancer Patients

**DOI:** 10.1155/2021/7647139

**Published:** 2021-02-17

**Authors:** Fangfang Chen, Shuai Yin, Bin Luo, Xiaoyan Wu, Honglin Yan, Dandan Yan, Chuang Chen, Feng Guan, Jingping Yuan

**Affiliations:** ^1^Department of Pathology, Renmin Hospital of Wuhan University, No. 99, Zhangzhidong Road, Wuchang District, Wuhan, 430060 Hubei, China; ^2^Department of Gastroenterology, Jingzhou Central Hospital, The Second Clinical Medical College, Yangtze University, Jingzhou, Hubei, China; ^3^Department of Breast and Thyroid Surgery, Renmin Hospital of Wuhan University, Wuhan, 430060 Hubei, China

## Abstract

**Aim:**

The main objective of this article was to evaluate the association of voltage-dependent anion channel 1 (VDAC1) with Cytochrome C (Cytc) expression, various clinicopathological features, and prognosis in breast cancer (BC) patients. Meanwhile, the correlation of Cytc expression with various clinical features and 5-year disease-free survival (5-DFS) of BC was also investigated.

**Methods:**

*In vivo*, expression of VDAC1 and Cytc was examined in 219 BC tissues and 100 benign breast lesions by immunohistochemical (IHC) analysis. *In vitro*, MTT and wound healing migration assay were performed to detect the effect of VDAC1 on BC cells.

**Results:**

Expression of VDAC1 is conversely associated with Cytc in BC (*P* = 0.011), especially in triple-negative breast cancer (TNBC) (*P* = 0.004). Knockdown of VDAC1 inhibited proliferation (*P* < 0.001) and migration (*P* < 0.05) of MCF-7 cells. High expression of VDAC1 and low expression of Cytc had a significant association with multiple clinicopathological parameters (*P* < 0.05) and poor 5-DFS (*P* < 0.001) in BC.

**Conclusion:**

VDAC1 was elevated in BC tissues and conversely associated with Cytc. Detection of VDAC1 may provide guidance for the poor prognosis of BC, especially TNBC.

## 1. Introduction

Breast cancer (BC) is by far the most common female malignant tumor in the world, with a high mortality rate, and is the leading cause of cancer-related death in women [1]. In China, 367900 new cases of BC were diagnosed in 2018, which accounts for 19.2% of all newly diagnosed female cancers [2]. In recent years, although breast conserving surgery, targeted therapy, endocrine therapy, and immunotherapy have made progress in breast cancer treatment, the incidence rate of the disease has increased [3]. And only limited success has been achieved in cases of advanced cancer [4]. The burden of this disease is heavy, and it remains a serious health threat for women relative to the large Chinese population [5]. Therefore, it is extremely important to find new prognostic markers and early diagnosis of BC, so that patients can receive better treatments.

Cancer cells have a series of common characteristics, including high proliferation and antiapoptosis [6, 7]. The formation of cancer is related to cell metabolic reprogramming, such as enhanced aerobic glycolysis (Warburg effect) [8, 9]. And the “Warburg effect” is postulated to be closely associated with the closure of voltage-dependent anion channel (VDAC) [10, 11]. VDAC has been identified as a 31 kDa pore-forming protein, which is present in the mitochondrial outermembrane (OMM) of all eukaryotes [12]. Three homologous genes encode three VDAC isoforms: VDAC1, VDAC2, and VDAC3 [13]. Among them, VDAC1 is the most abundantly expressed and best characterized one, which forms a channel for the entry and exit of mitochondrial metabolites including ATP and NADH across the OMM, thereby regulating the activity of mitochondria [14]. VDAC1 plays a key role in the regulation of apoptosis by both interaction with proapoptotic (Bcl2 and Bcl-xL) proteins and antiapoptotic proteins (Bax, Bak, and Bim) [15, 16]. It has been proved that VDAC1 is involved in several diseases, such as neurodegenerative, cardiac injury, and neoplastic [17]. Several studies have demonstrated that VDAC1 is remarkable expressed in malignant tumors such as uterine cervical cancer [18], hepatocellular carcinoma [19], and cholangiocellular carcinoma [20], indicating that it plays a significant role in high energy-demanding cancer cells. Furthermore, VDAC1 has also been reported to promote tumor growth and play a controversial role in the prognosis of different cancers [21]. A number of anticancer agents have taken VDAC1 as a novel pharmacological target [12]. However, the correlation of VDAC1 expression with tumorigenesis and progression of solid tumor of BC and the role of VDAC1 acts in the prognosis of BC patients has not been well-documented.

Cytochrome C (Cytc) has been proposed to be a proapoptotic factor which is located in the inner membrane of mitochondria and plays an irreplaceable role in mitochondrial electron transport and intrinsic type II apoptosis [22, 23]. Cytc release forms an essential step in the apoptotic cascade, upon binding with apoptosis protease-activating factor 1 (Apaf-1), dATP, and procaspase-9 [24, 25]. A number of studies have shown that Cytc can induce apoptosis of cancer cells [26, 27]. Jamsheed et al. [28] demonstrated that in non-small-cell lung cancer patients, Cytc level was lower than healthy individuals, and the lower expression of Cytc were associated with advanced stages, high grade histological differentiation, and shorter overall survival. Rahul et al. [29] explored that in prostate cancer, the deficiency of Cytc contributed to tumor invasiveness and therapeutic resistance and led to faster recurrence. It has been widely reported that VDAC1 is involved in the release of Cytc, which is able to signal Cytc to initiate the mitochondrial-mediated cell death cascade [30]. In contrast, VDAC1 has also been reported to interact with antiapoptotic proteins such as Bcl-2 and hexokinase (HK) to control the release of Cytc [15]. In several melanoma and prostate cancer cell lines, the expression level of VDAC1 is related to the induction of Cytc release, which provides more possibilities for the pharmacological treatment of tumors [31]. However, the association of VDAC1 expression with Cytc in BC is still elusive and rarely addressed, thereby indicating further investigation is needed.

The main objectives of this article were as follows: (i) evaluate the association of VDAC1 expression with Cytc in BC; (ii) investigate the correlation of various clinical features and 5-year disease-free survival (5-DFS) of BC with VDAC1 and Cytc, respectively, and (iii) explore the effects of VDAC1 on cell proliferation and migration in BC cell line.

## 2. Material and Methods

### 2.1. Patient Tissue Samples

A total of 219 formalin-fixed, paraffin-embedded primary invasive breast cancer tissue samples were collected from the patients diagnosed with BC through histopathologic evaluation on surgical tissue specimens. 100 cases of benign breast lesions were collected as controls. All the patients underwent surgical treatment at Renmin Hospital of Wuhan University between August 2009 and December 2010. There were no any previous chemotherapies, radiotherapies, or other treatments before surgery in these patients. The patients' written informed consent was obtained before the operation, and the study was approved by the Ethics Committee of Renmin Hosptial of Wuhan University. Patients were all followed up for 5 years. The follow-up data was calculated as the period from the date of surgery to the end of follow-up or death. We followed up all the patients by telephone interviews or outpatient clinic visits.

### 2.2. Cell Culture

The breast cancer cell line MCF-7 was purchased from the Cell Bank of Chinese Academy of Sciences (Shanghai, China). These cells were incubated in Dulbecco's modified Eagle's medium (DMEM, Invitrogen, Carlsbad, CA, USA) supplemented with 10% fetal bovine serum (FBS, Invitrogen), routinely maintained at 37°C and in an atmosphere of 5% CO2.

### 2.3. Immunohistochemistry (IHC)

A tissue array was used which included two tumor samples from each patient. The paraffin-embedded tissues were cut into 4 *μ*m thick sections, then deparaffinized and dehydrated following standard procedures. Subsequently, paraffin sections were rinsed with PBS (3 × 5 min) and then blocked with 3% hydrogen peroxide at 37°C for endogenous peroxidase ablation for 10 min. Antigen retrieval was conducted by microwave heating with citrate buffer (pH 6.0) for 20 min. Then, the samples were exposed to normal goat serum at 37°C for 20 min to decrease nonspecific antibody binding. The tissue sections were incubated overnight at 4°C with the primary antibody (anti-VDAC1, 1 : 1000, ab15895, Abcam, UK; anti-Cytc, 1 : 50, sc-13561, Santa Cruz Biotechnology, Inc., Santa Cruz, CA, USA). After rinsing in PBS, the tissue sections were incubated with horseradish peroxidase-labeled anti-rabbit antibodies at 37°C for 20 min. Then, the tissue sections were rinsed with PBS for 4 times and then dripped with freshly prepared 3,3-diaminobenzidine (DAB). Microscopically, the staining was terminated when the tissue sections were brown-yellow or brown. Subsequently, all the tissue sections were restained with hematoxylin for about 1 min. Finally, the slices were dehydrated with ethanol and toluene and then sealed with neutral gum. PBS was used to replace the primary antibody as negative control.

### 2.4. Evaluation of Immunohistochemical Staining

The slides were viewed via Olympus BX53 (Tokyo, Japan) microscope. IHC staining was evaluated independently by two pathologists under the double-blind condition. VDAC1 was mainly expressed in membrane of tumor cells. VDAC1 immunohistochemical staining in tumor cells was evaluated semiquantitatively as follows: (1) staining intensity: 0 (no staining), 1 (weak staining), 2 (moderate staining), and 3 (strong staining); (2) the extent of staining: 0 (0%), 1 (1-20%), 2 (21-50%), and 3 (>50%). Five most representative fields of high magnification (400x) were selected to calculate the final score. The final immunohistochemical score was multiplied by staining intensity and extent, theoretically from 0 to 9. Scores less than or equal to 3 were defined as low expression, and scores greater than or equal to 4 were described as high expression. Cytc protein was predominantly located in membrane and cytoplasm of tumor cells. The staining intensity was classified as four grades as follows: 0 (no staining), 1 (light yellow), 2 (brown-yellow), and 3 (dark brown). The percentage of positive cells was classified as five grades as follows: 0 (0%), 1 (≤30%), 2 (31-50%), 3 (51-80%), and 4(≥80%). Five most representative fields of high magnification (400x) were selected to calculate the final score. The final immunohistochemical score was the product of staining intensity and extent, theoretically from 0 to 12. Scores less than 4 were defined as low expression, and scores greater than or equal to 4 were described as high expression.

### 2.5. Transfection

Small interfering RNA (siRNA) duplexes targeting human VDAC1 (si-VDAC1-1: GTCTAGGACTGGAATTTCA, si-VDAC1-2: GGAGACCGCTGTCAATCTT, and si-VDAC1-3: GCTGCGACATGGATTTCGA) were designed and synthesized by Guangzhou Ruibo Biotechnology Co., Ltd (Guangzhou, China). SiRNA duplexes with nonspecific sequences were used as siRNA negative control. The transfections were carried out using Lipofectamine-RNAi MAX (Invitrogen) following the manufacturer's protocol. The siRNA-targeted human VDAC1 is designed from the messenger RNA (mRNA) sequences of human VDAC1 gene (RefSeq: NM_003374). The effect of silencing was verified at the protein level.

### 2.6. Western Blot Analysis

Cultured cells were collected and lysed to harvest protein. Denatured protein samples were electrophoretically separated on 10% SDS-polyacrylamide gel (PAGE), and then, the proteins were transferred to polyvinylidenefluoride (PVDF) membranes. After transfer, the membranes were incubated with a blocking buffer consisting of 50 mm Tris-HCl (pH 7.5), 100 mm NaCl, 5% nonfat dry milk, and 0.05% Tween 20 for 2 h. Next, the membranes were incubated with primary antibodies against VDAC1 (Abcam Ab15895, 1 : 1000) and Cytc (Santa Cruz sc-13561, 1 : 100) at 4°C overnight. After being washed for three times, the membranes were further incubated with horseradish peroxidase-conjugated sheep antihuman (Amersham Biosciences, Piscataway, NJ) IgG antibodies at a dilution of 1 : 5000 for 2 h at room temperature. Protein was visualized using an enhanced chemiluminescence system (ECL) reagent (KeyGEN BioTECH, China). The volumes of target bands were normalized to GAPDH. Then, the membranes were detected by automatic chemiluminescence analyzer, and the gray value of related bands was analyzed by Image J software (Broken Symmetry Software). Each experiment was repeated at least three times.

### 2.7. MTT Assay

Cell proliferation viability of MCF-7 cells was measured using the MTT assay. In brief, cells were seeded after transfection with siRNA for the indicated time in triplicate in 96-well plates at densities of 3 × 10^3^ cells/well. The cells were incubated under standard conditions for 48 hours. Then, cells were treated by MTT dye (5 mg/ml 10 *μ*l per well) for 4 h. The absorbance values were measured using a microplate reader at a wavelength of 490 nm. The growth assays were repeated three times and reported as percentage changes compared to the controls.

### 2.8. Wound Healing Migration Assay

A total of 1 × 106 cells were plated into a 6-well plate, and complete convergence was allowed. Wounds were created by scraping confluent cell monolayers with 200 *μ*l pipette tips. The cells were washed with PBS twice, after which cells were cultured in 10% FBS DMEM. Three digital images were taken at 0 and 48 h after wounding under a microscope (Olympus, Japan). The assays were accomplished in triplicate and repeated three times. The change of wound width was measured, and wound area filled was calculated by Image J software (Broken Symmetry Software).

### 2.9. Statistical Analysis

SPSS software version 17.0 was used to carry out all the statistical analyses. Chi-square test and Student's *t* test were used to analyze the association between categorical variables. Spearman's rank correlation analysis performed to evaluate correlations between variables. The survival curves were disposed by using the Kaplan-Meier method and log-rank test. We performed univariate and multivariate survival analysis through Cox proportional hazard regression model to assess the independent prognostic factors in BC patients. Hazard ratios (HRs) and their 95% confidence intervals (CIs) were calculated for both univariate and multivariate analyses. Two-tailed *P* values of <0.05 were considered statistically significant. Graph-Pad Prism 8.0.1 (GraphPad, San Diego, CA) software was used to present graphs.

## 3. Results

### 3.1. The Characteristics of Patients

The patients were composed of 219 females with a median age of 50 (age range, 29–78) years. Among the 219 cases, 165 (75.34%) were classified into G1 and G2 stages, 54 (24.66%) were defined with G3 on the basis of histological differentiation. 153 (69.86%) patients were classified as stages I and II, and 66 (30.14%) as III on the basis of tumor node metastasis (TNM) stage. Other basic clinicopathological characteristics, including age, menopausal status, lymph node metastasis (LNM), estrogen receptor (ER), progesterone receptor (PR), human epidermal growth factor receptor 2 (HER2), and recurrence, were presented in Table 1.

### 3.2. Expression of VDAC1 Is Significantly Higher in BC Tissues than in Benign Breast Lesions

**Figure 1:**
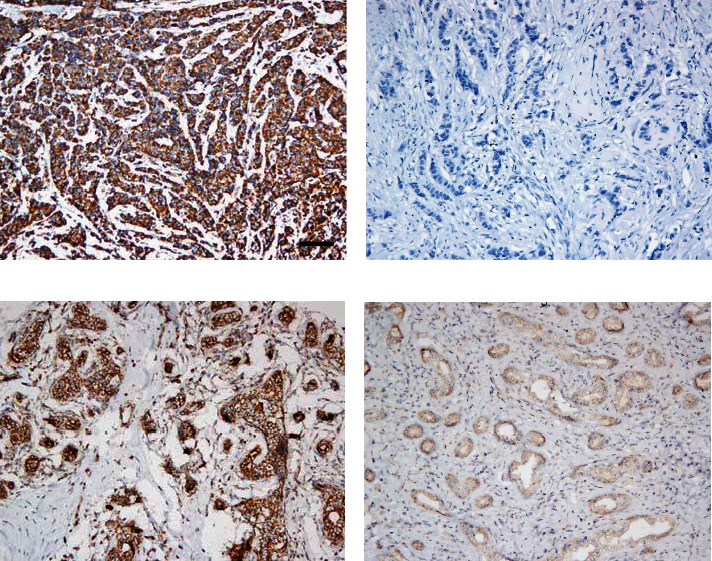
Immunohistochemical staining of VDAC1 in breast cancer (BC) lesions and benign breast lesions: (a) high expression of VDAC1 protein in BC; (b) low expression of VDAC1 protein in BC; (c) high expression of VDAC1 protein in benign breast lesions; (d) low expression of VDAC1 protein in benign breast lesions. Scale bar, 50𝜇m.

To examine the expression level of VDAC1 protein, we performed IHC on 219 cases of BC tissues and 100 cases of the benign breast lesions. As shown in Figure 1, VDAC1 protein was mainly expressed in the membrane of breast cancer cells.  Table 2 showed the result of IHC staining of VDAC1 protein. Of 219 BC samples, 162 (73.97%) showed high expression of VDAC1 protein, and 57 (26.03%) showed low expression. In benign breast lesions, 55 (55%) showed high expression of VDAC1 protein, and 45 (45%) showed low expression. The expression of VDAC1 was significantly higher in BC tissues (*χ*^2^ = 11.361, *P* = 0.001) as determined by chi-square test.

### 3.3. Expression of Cytc Is Lower in BC Tissues than in Benign Breast Lesions

**Figure 2:**
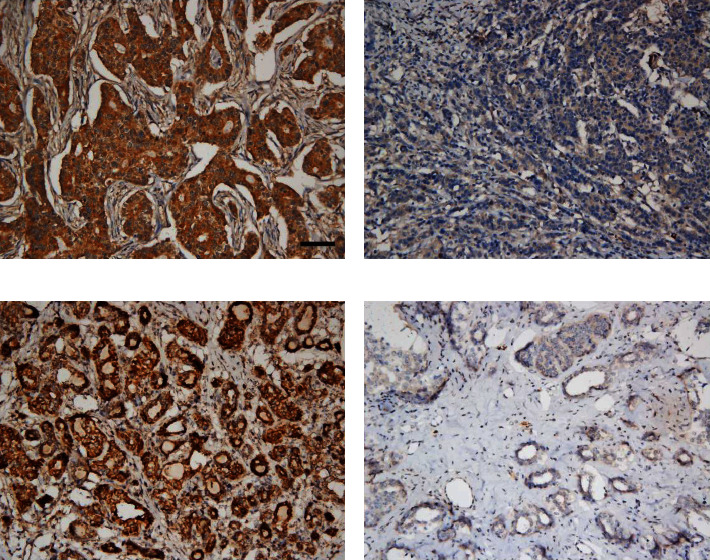
Immunohistochemical staining of Cytc in BC lesions and benign breast lesions: (a) high expression of Cytc protein in BC; (b) low expression of Cytc protein in BC; (c) high expression of Cytc protein in benign breast lesions; (d) low expression of Cytc protein in benign breast lesions. Scale bar, 50𝜇m.

We examined the expression of Cytc by IHC in 219 cases of BC tissue and 100 cases of the benign breast lesions. As shown in Figure 2, Cytc protein was mainly expressed in the membrane and cytoplasm of BC tumor cells. The result of IHC staining of Cytc protein was summarized in Table 2, which showed that the rate of high Cytc expression was 29.68% (65/219) and the rate of low Cytc expression was 70.32% (154/219) in all BC samples. While in benign breast lesions, the rate of high Cytc expression was 47% (47/100), and the rate of low Cytc expression was 53% (53/100). Thus, the expression of Cytc was significantly lower in BC tissues than benign breast lesions (*χ*^2^ = 9.039, *P* = 0.004).

### 3.4. VDAC1 Protein Expression Correlates with Cytc Protein Expression and Clinicopathological Parameters in BC Tissues, but Not in Benign Breast Lesions

**Figure 3:**
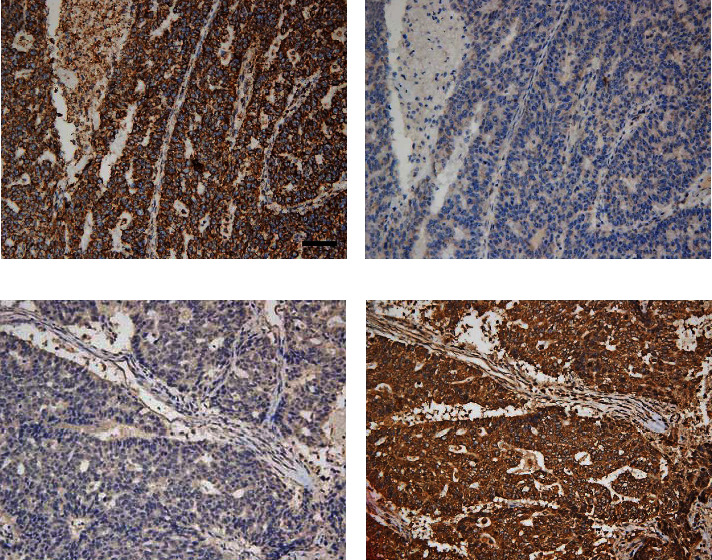
Immunohistochemical staining for VDAC1 and Cytc in breast cancer tissues: high expression of VDAC1 protein (a) with low Cytc expression (b); low expression of VDAC1 protein (c) with high Cytc expression (d). Scale bar, 50𝜇m.

In the benign breast lesions, there was no significant association between VDAC1 expression and Cytc protein expression (*χ*^2^ = 0.75, *P* = 0.425; Table 3). Conversely, as shown in Table 4, high expression of VDAC1 protein was inversely associated with Cytc protein expression in BC tissues (*χ*^2^ = 7.423, *r* = −0.184, *P* = 0.011), which was also shown in Figure 3. Furthermore, as shown in Table 5, high expression of VDAC1 protein was detected in 73.97% (162/219) of BC tissues, which was significantly associated with advanced TNM stage (*χ*^2^ = 7.534, *P* = 0.007), higher histological grade (*χ*^2^ = 4.68, *P* = 0.033), recurrence (*χ*^2^ = 24.532, *P* < 0.001), HER2 gene amplification (*χ*^2^ = 6.949, *P* = 0.008), and lymph node metastasis (*χ*^2^ = 5.109, *P* = 0.03), but not with other examined clinicopathological parameters, including age (*χ*^2^ = 0.348, *P* = 0.636), ER status (*χ*^2^ = 0.729, *P* = 0.44), PR status (*χ*^2^ = 0.499, *P* = 0.535), and menopause (*χ*^2^ = 0.028, *P* = 0.878).

### 3.5. Cytc Expression Is Also Correlated with Various Clinical Features in BC Tissues

As is shown in Table 5, low expression of Cytc was associated with higher histological grade (*χ*^2^ = 4.278, *P* = 0.041), ER status (*χ*^2^ = 4.609, *P* = 0.037), PR status (*χ*^2^ = 5.424, *P* = 0.025), and recurrence (*χ*^2^ = 8.629, *P* = 0.004), but not age (*χ*^2^ = 0.106, *P* = 0.763), TNM stage (*χ*^2^ = 0.018, *P* = 0.895), lymph node metastasis (*χ*^2^ = 0.003, *P* = 0.953), HER2 gene amplification (*χ*^2^ = 0.36, *P* = 0.591), or menopause (*χ*^2^ = 0.751, *P* = 0.455) in tumor samples.

### 3.6. Expression Level of Cytc Protein Is Increased after VDAC1 Knockdown In Vitro

**Figure 4:**
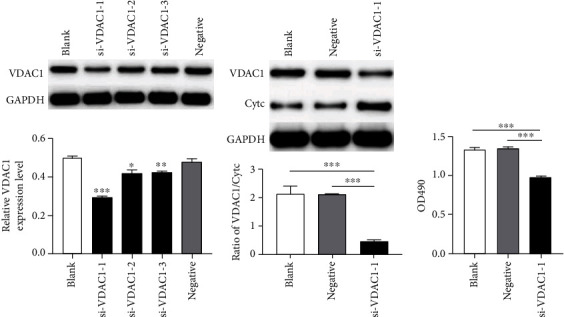
(a) VDAC1 expression was examined by Western blot in MCF-7 cells transfected with three different siRNAs targeting VDAC1 (si-VDAC1-1, siVDAC1-2, and si-VDAC1-3). (b) VDAC1 and Cytc expressions were examined by Western blot in MCF-7 cells transfected with si-VDAC1-1. After standardization with GADPH, the ratio of VDAC1/Cytc was calculated. (c) Proliferation of MCF-7 cells was demonstrated by MTT assay after knockdown of VDAC1. Each value represents the mean ± SD; ^∗^*P* < 0.05, ^∗∗^*P* < 0.01, ^∗∗∗^*P* < 0.001.

Firstly, we conducted transient silencing of VDAC1 in the MCF-7 cell line with three different siRNAs and verified the knockdown efficiency with Western blot. Si-VDAC1-1 achieved more effective knockdown efficiency, so it was used in all subsequent experiments (*P* < 0.001, Figure 4(a)).

As VDAC1 was conversely associated with Cytc expression in BC tissues, we also explored the expression level of Cytc *in vitro* after knockdown of VDAC1. Consistently, silencing of VDAC1 led to Cytc expression upregulation at the protein level in MCF-7 cells (*P* < 0.001, Figure 4(b)).

### 3.7. VDAC1 Promoted the Proliferation and Migration of BC Cells In Vitro

**Figure 5:**
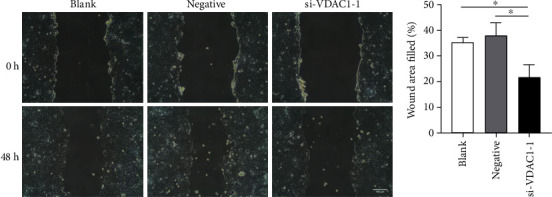
(a, b) Effects of VDAC1 knockdown on MCF-7 cells migration were assessed using a wound healing migration assay after 0 h and 48 h. Each value represents the mean ± SD; ^∗^*P* < 0.05, ^∗∗^*P* < 0.01, ^∗∗∗^*P* < 0.001.

Additionally, we explored whether VDAC1 could affect the cell proliferation and migration. After transfection with si-VDAC1-1, a significant reduction in the proliferation rate of MCF-7 cells was observed with the MTT assay compared with the control (*P* < 0.001, Figure 4(c)). Afterwards, wound healing migration assay was performed. As a result, we validated that VDAC1 silencing obviously inhibited MCF-7 cell migration compared with the controls (*P* < 0.05, Figure 5). Therefore, knockdown of VDAC1 can suppress the proliferative and migrative ability of MCF-7 cells *in vitro*.

### 3.8. Correlation Analysis of the 5-DFS with the Expression of VDAC1, Cytc Protein, and Other Parameters in BC

**Figure 6:**
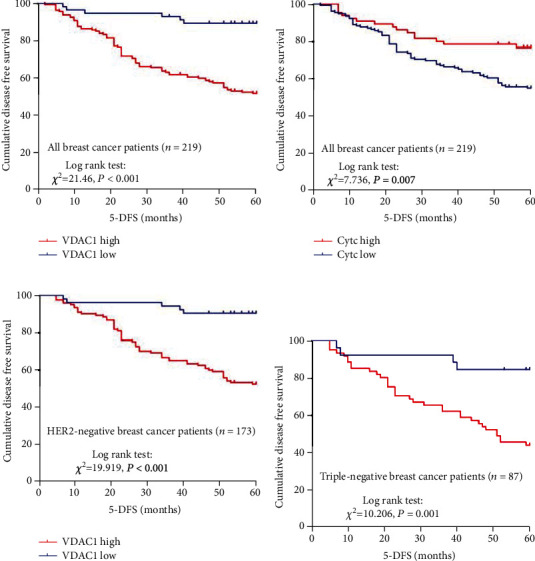
Kaplan-Meier survival analysis showing the correlation between VDAC1 expression (a), Cytc expression (b), and 5-DFS in breast cancer patients; the correlation between VDAC1 expression and 5-DFS in HER2-negative breast cancer (c) and triple-negative breast cancer (d) (log-rank test).

Kaplan-Meier analysis showed that low VDAC1 protein expression in BC predicted a better survival and lower mortality rate (Figure 6(a), *P* < 0.001). Similarly, high expression of Cytc also predicted a better outcome of BC patients (Figure 6(b), *P* = 0.007). Univariate analysis of predictive factors for the 5-DFS in BC patients was performed by Cox proportional hazards regression model (Table 6). In univariate analysis, histological grade (*P* < 0.001), TNM stage (*P* < 0.001), ER status (*P* = 0.033), PR status (*P* = 0.001), and lymph node metastasis (*P* < 0.001) were also significantly correlated with the 5-DFS of BC patients. However, age, HER2 gene amplification, and menopause had no significant association with 5-DFS in BC patients (*P* > 0.05) (Table 6). In order to analyze whether the above univariate was an independent prognostic factor, multivariate Cox proportional hazard model for 5-DFS was performed. The results indicated that both the expressions of VDAC1 and Cytc were independent prognostic parameters for 5-DFS of BC patients [HR: 3.982 (1.723-9.207), *P* = 0.001; HR: 0.542 (0.307-0.959), *P* = 0.035, respectively, Table 6]. Concurrently, TNM stage, PR status, lymph node metastasis, and histological grade were also independent predictors regarding the 5-DFS of breast cancer patients.

### 3.9. High Expression of VDAC1 Is Associated with the Poor Prognosis of HER2-Negative Breast Cancer

**Figure 7:**
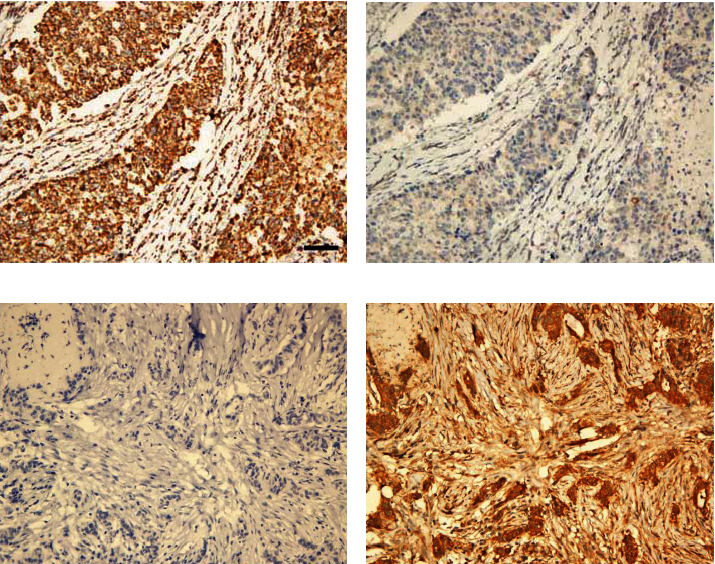
Immunohistochemical staining for VDAC1 and Cytc in triple-negative breast cancer lesions: high expression of VDAC1 protein (a) with low Cytc expression (b); low expression of VDAC1 protein (c) with high Cytc expression (d). Scale bar, 50𝜇m.

As is shown in Table 5, the expression of VDAC1 was significantly associated with HER2 gene, but not with ER and PR status. As targeted therapy can be implemented for HER2-positive breast cancer, we analyzed the correlation of VDAC1 protein expression with the prognosis of HER2-positive breast cancer patients and HER2-negative breast cancer patients, respectively. Kaplan-Meier analysis showed that VDAC1 expression had no correlation with 5-DFS in HER2-positive breast cancer patients (*P* = 0.159). However, a significant relevance was found between high VDAC1 expression and shorter 5-DFS (*P* < 0.001) in HER2-negative breast cancer patients (Figure 6(c)). As triple-negative breast cancer (TNBC) is a special molecular subtype of HER2-negative breast cancer, which is defined by the absence of the ER, PR, and HER2 genes, with no standard treatment at present, we then explored the correlation of VDAC1 expression with the prognosis of TNBC. Interestingly, VDAC1 was also associated with reduced 5-DFS in TNBC (*P* = 0.001) (Figure 6(d)), predicting a poorer survival and higher mortality rate. To further validate the prognostic significance of VDAC1, Cox proportional hazards model analysis was also performed in TNBC. As presented in Table 7, VDAC1 was an independent predictor of poor 5-DFS [HR: 4.018 (1.415-11.407), *P* = 0.009]. Meanwhile, VDAC1 protein expression was inversely associated with Cytc in TNBC (*χ*^2^ = 9.102, *r* = −0.323, *P* = 0.004, Table 8, Figure 7). In summary, high expression of VDAC1 is associated with the prognosis of TNBC and inversely correlates with Cytc.

## 4. Discussion

In this study, we observed that VDAC1 was elevated while Cytc was decreased in breast carcinoma patients compared with benign breast lesions. VDAC1 protein expression was conversely associated with Cytc in BC, especially in TNBC. Meanwhile, knockdown of VDAC1 inhibits BC cell proliferation and migration *in vitro*. Furthermore, both high VDAC1 and low Cytc protein expression had significant positive correlation with poor prognosis. VDAC1 expression can also be an independent prognostic factor of BC, especially TNBC.

VDAC1 participates in cancer metabolism via its modulatory roles in the transport of various metabolites [32]. In many types of cancer, the interaction of VDAC1 with HK, especially HK II, directly accesses to mitochondrial ATP for phosphorylation of glucose to glucose-6-phosphate and contributes to the cancer cells unrestricted growth and the inhibiting of apoptosis [21, 33]. VDAC1 has been found to be involved in tumor proliferation, migration, metastasis, and invasion [13]. It has been demonstrated that VDAC1 acts a controvertial role in the prognosis of different malignant tumors. For example, in uterine cervical cancer, high expression of VDAC1 was associated with exhibited deeper stromal invasion, larger tumor size, higher recurrence, and poorer overall survival [18]. Conversely, in cholangiocellular carcinoma, the low expression of VDAC1 correlated with higher cancer stage classification, lymph node involvement, and reduced survival [20]. These conflicting consequences demonstrate the differential effects of VDAC1 expression in different kinds of cancer and may need further exploration. In the present study, 219 cases of primary invasive breast cancer tissues were collected, and it was found that VDAC1 protein was primarily located in the membrane of breast cancer cells. The expression of VDAC1 protein in breast cancer solid tumors was significantly higher than that in benign breast lesions, and high expression of VDAC1 protein correlated with advanced TNM stage, higher histological grade, recurrence, lymph node metastasis, and HER2 gene amplification, thereby suggesting that high VDAC1 expression may play a role in promoting tumorigenesis and progression of BC. Interestingly, our present results in breast cancer were consistent with the reports in pancreatic cancer [34] and colorectal cancer [35], which indicated VDAC1 expression was upregulated in tumor and promoted the growth and invasion of cancer cells. In addition, knockdown of VDAC1 in multiple types of cancer cell lines, including colon and lung cancer cells, has been demonstrated to block proliferation and migration of the cancer cells *in vitro* [36–38]. As is shown above, our studies achieved the similar results.

Furthermore, by the multivariate analysis, we found that overexpression of VDAC1 protein in BC tissues could be as an independent poor prognostic factor. Consistent with this result, Chih-Hsien and Eiran's studies also showed that high expression of VDAC1 was correlated with poorer prognosis in uterine cervical cancer and hepatocellular carcinoma [18, 19]. Therefore, these data suggest that VDAC1 has the potential to be a poor prognostic marker in BC. As TNBC showed more progressively malignant manifestation with worse clinical outcomes and is the most insensitive subtype of breast cancer to drug treatment, we next explored the correlation of VDAC1 expression with the prognosis of TNBC. Amazingly, our result demonstrated that high level of VDAC1 protein was also associated with reduced 5-DFS and acted as an independent predictor of poor prognosis in TNBC, suggesting more potential use of VDAC1 should be exploited in prognostic marker and therapeutic target.

Cytc release from mitochondria is the driving force for apoptosome leading to apoptotic cell death in several malignant tumor [39], and VDAC1 has been widely reported to be interacted with pro- or antiapoptotic proteins such as Bcl-2 and HK, whereby mediating the release of Cytc [15, 40]. In many types of cancer cells, when HK2 binds to VDAC1, the interaction between VDAC1 and Bcl-2 protein family will be blocked, resulting in a decrease in Cytc release, thus protecting tumor cells from apoptosis [21]. In our present study, VDAC1 protein expression was inversely associated with Cytc in BC, especially in TNBC. Simultaneously, silencing of VDAC1 upregulated Cytc expression at the protein level in MCF-7 cells. Furthermore, Cytc was lower expressed in BC compared with benign breast lesions and low expression of Cytc was correlated with higher histological grade, ER status, PR status, and recurrence. A similar correlation was found in prostate cancer which Cytc deficiency contributed to tumor invasiveness and faster recurrence [29]. Importantly, we also found that decreased expression of Cytc was an independent prognostic factor and played a pivotal role in the poorer 5-DFS of that cohort of BC patients. As a result, contrary to VDAC1, Cytc has the potential to be an improved prognostic marker in BC.

It should be acknowledged that there are also some limitations in this study. Firstly, due to the limited number of patients in this study, a larger cohort is required. Secondly, we took the benign breast tissues as control, which was not paired comparison, so associations should be interpreted with cognizance of these possible differences. Besides, more investigations need to be conducted to explore the mechanisms by which VDAC1 reduces Cytc expression in BC. Afterwards, the prognostic significance of VDAC1 in BC was discussed only at histological level. The exact role of VDAC1 in BC, especially in TNBC, still needs to be evaluated in follow-up mechanistic investigations. Finally, our study evaluated prognosis by 5-DFS rather than OS. Since DFS was not correlated with OS sometimes, the influence of VDAC1 expression on OS is still a topic for future research.

## 5. Conclusion

Our study showed for the first time that VDAC1 was elevated in BC tissues. Meanwhile, our findings firstly revealed that VDAC1 expression was conversely associated with Cytc, and knockdown of VDAC1 inhibited malignant behavior of BC. Besides, high VDAC1 level was associated with reduced 5-DFS of BC patients. By the multivariate analysis, we found that overexpression of VDAC1 could be employed as an independent poor prognostic factor in breast cancer, including TNBC, which was intractable in clinical. All in all, VDAC1 can be exploited as a potential prognostic marker and therapeutic target in BC, especially TNBC.

## Figures and Tables

**Table 1:** Patient characteristics (*N* = 219).

Characteristic	*N* (%)
Age (years)	
≤50	135 (61.64%)
>50	84 (38.36%)
Menopause	
Before	125 (57.08%)
After	94 (42.92%)
TNM stage	
I/II	153 (69.86%)
III	66 (30.14%)
Histological grade	
G1/G2	165 (75.34%)
G3	54 (24.66%)
Lymph node metastasis	
No	95 (43.38%)
Yes	124 (56.62%)
ER status	
Negative	122 (55.71%)
Positive	97 (44.29%)
PR status	
Negative	124 (56.62%)
Positive	95 (43.38%)
HER2 gene	
Nonamplification	173 (79%)
Amplification	46 (21%)
Recurrence	
No	136 (62.1%)
Yes	83 (37.9%)

**Table 2:** The expression of VDAC1 and Cytc protein in breast cancer and benign breast lesions.

	VDAC1		*P* value	Cytc		*P* value
	High expression	Low expression		High expression	Low expression	
Breast cancer tissue (*n* = 219)	162 (73.97%)	57 (26.03%)	**0.001**	65 (29.68%)	154 (70.32%)	**0.004**
Benign breast lesions (*n* = 100)	55 (55%)	45 (45%)		47 (47%)	53 (53%)	

**Table 3:** Correlation analysis of the expression of VDAC1 with Cytc in benign breast lesions.

	High-VDAC1 expression (*n* = 55)	Low-VDAC1 expression (*n* = 45)	*P* value
Cytc expression			
High (*n* = 47)	28 (59.57%)	19 (40.43%)	0.425
Low (*n* = 53)	27 (50.94%)	26(49.06%)	

**Table 4:** Correlation analysis of the expression of VDAC1 with Cytc in breast cancer patients.

	High-VDAC1 expression (*n* = 162)	Low-VDAC1 expression (*n* = 57)	*r* value	*P* value
Cytc expression				
High (*n* = 65)	40 (61.54%)	25 (38.46%)	**-0.184**	**0.011**
Low (*n* = 154)	122 (79. 22%)	32 (20.78%)		

**Table 5:** Correlation between the expression of VDAC1, Cytc, and clinicopathologic parameters.

	*n*	VDAC1		*P* value	Cytc		*P* value
		High (*n* = 162)	Low (*n* = 57)		High (*n* = 65)	Low (*n* = 154)	
Age				0.636			0.763
≤50	135	98 (72.59%)	37 (27.41%)		39 (28.89%)	96 (71.11%)	
>50	84	64 (76.19%)	20 (23.81%)		26 (30.95%)	58 (69.05%)	
Menopause				0.878			0.455
Before	125	93 (74.4%)	32 (25.6%)		40 (32%)	85 (68%)	
After	94	69 (73.4%)	25 (26.6%)		25 (26.6%)	69 (73.4%)	
Recurrence				**0.000**			**0.004**
No	136	85 (62.5%)	51 (37.5%)		50 (36.76%)	86 (63.24%)	
Yes	83	77 (92.77%)	6 (7.23%)		15 (18.07%)	68 (81.93%)	
TNM stage				**0.007**			0.895
I/II	153	105 (68.63%)	48 (31.37%)		45 (29.41%)	108 (70.59%)	
III	66	57 (86.36%)	9 (13.64%)		20 (30.3%)	46 (69.7%)	
Lymph node metastasis				**0.030**			0.953
No	95	63 (66.32%)	32 (33.68%)		28 (29.47%)	67 (70.53%)	
Yes	124	99 (79.84%)	25 (20.16%)		37 (29.84%)	87 (70.16%)	
Histological grade				**0.033**			**0.041**
G1/G2	165	116 (70.3%)	49 (29.7%)		55 (33.33%)	110 (66.67%)	
G3	54	46 (85.19%)	8 (14.81%)		10 (18.52%)	44 (81.48%)	
HER2 gene				**0.008**			0.591
Nonamplification	173	121 (69.94%)	52 (30.06%)		53 (30.64%)	120 (69.36%)	
Amplification	46	41 (89.13%)	5 (10.87%)		12 (26.09%)	34 (73.91%)	
ER status				0.440			**0.037**
Negative	122	93 (76.23%)	29 (23.77%)		29 (23.77%)	93 (76.23%)	
Positive	97	69 (71.13%)	28 (28.87%)		36 (37.11%)	61 (62.89%)	
PR status				0.535			**0.025**
Negative	124	94 (75.81%)	30 (24.19%)		29 (23.39%)	95 (76.61%)	
Positive	95	68 (71.58%)	27 (28.42%)		36 (37.89%)	59 (62.11%)	

**Table 6:** Univariate and multivariate analyses of predictive factors for disease free survival in BC patients.

	*n*	Univariate		Multivariate	
		*P* value	Hazard ratio, 95% CI	*P* value	Hazard ratio, 95% CI
VDAC1 expression		**0.000**	5.636 (2.454-12.942)	**0.001**	3.982 (1.723-9.207)
High	162				
Low	57				
Cytc expression		**0.007**	0.463 (0.265-0.811)	**0.035**	0.542 (0.307-0.959)
High	65				
Low	154				
Age		0.718	1.084 (0.699-1.683)		
≤50	135				
>50	84				
TNM stage		**0.000**	3.879 (2.512-5.989)	**0.028**	1.772 (1.063-2.956)
I/II	153				
III	66				
Lymph node metastasis		**0.000**	4.717 (2.692-8.265)	**0.003**	2.690 (1.401-5.164)
No	95				
Yes	124				
Histological grade		**0.000**	5.399 (3.489-8.356)	**0.000**	2.998 (1.899-4.731)
G1/G2	165				
G3	54				
HER2 gene		0.075	1.568 (0.956-2.574)		
Nonamplification	173				
Amplification	46				
ER status		**0.033**	0.613 (0.390-0.962)		
Negative	122				
Positive	97				
PR status		**0.001**	0.442 (0.275-0.711)	**0.016**	0.553 (0.341-0.896)
Negative	124				
Positive	95				
Menopause					
Before	125	0.184	1.339 (0.870-2.060)		
After	94				

**Table 7:** Univariate and multivariate analyses of predictive factors for disease free survival in TNBC.

	*n*	Univariate		Multivariate	
		*P* value	Hazard ratio, 95% CI	*P* value	Hazard ratio, 95% CI
VDAC1 expression		**0.001**	4.616 (1.635-13.029)	**0.009**	4.018 (1.415-11.407)
High	61				
Low	26				
Age		0.802	0.921 (0.484-1.753)		
≤50	49				
>50	38				
TNM stage		**0.000**	3.332 (1.757-6.317)	**0.003**	2.663 (1.381-5.135)
I/II	61				
III	26				
Lymph node metastasis		**0.002**	3.186 (1.504-6.747)		
No	38				
Yes	49				
Histological grade		**0.000**	3.149 (1.660-5.974)	**0.007**	2.469 (1.28-4.762)
G1/G2	59				
G3	28				
Menopause		0.590	0.839 (0.444-1.587)		
Before	43				
After	44				

**Table 8:** Correlation analysis of the expression of VDAC1 with Cytc in TNBC.

	High-VDAC1 expression (*n* = 61)	Low-VDAC1 expression (*n* = 26)	*r* value	*P* value
Cytc expression				
High (*n* = 19)	8 (24.24%)	25 (75.76%)	**-0.323**	**0.004**
Low (*n* = 68)	53 (77.94%)	15 (22.06%)		

## Data Availability

The datasets used and analyzed during the current study are available from the corresponding authors on reasonable request.
